# Neural Correlates of Deficits in Humor Appreciation in Gelotophobics

**DOI:** 10.1038/srep34580

**Published:** 2016-10-03

**Authors:** Yu-Chen Chan

**Affiliations:** 1 Institute of Learning Sciences, National Tsing Hua University, Hsinchu, Taiwan

## Abstract

Gelotophobics have social deficits in the form of relative humorlessness and heightened sensitivity to aggressive humor; however, little is known about the neural reward mechanisms for this group. The present study attempted to identify the neural substrates of responses to hostile and non-hostile jokes in gelotophobics and non-gelotophobics. Gelotophobics showed greater activation than did non-gelotophobics in the *dorsal corticostriatal system*, which comprises the dorsolateral prefrontal cortex and dorsal striatum, suggesting a higher degree of voluntary top-down cognitive control of emotion. As expected, gelotophobics showed less activation in the *ventral mesocorticolimbic system* (MCL) in response to both hostile and non-hostile jokes, suggesting a relative deficit in the reward system. Conversely, non-gelotophobics displayed greater activation than gelotophobics did in the MCL system, particularly for non-hostile jokes, which suggests a more robust bottom-up emotional response. In response to non-hostile jokes, non-gelotophobics showed greater activation in the ventral MCL reward system, which comprises the midbrain, amygdalae, nucleus accumbens, ventral anterior cingulate cortex, and insula. Psychophysiological interaction analyses further showed that gelotophobics exhibited diminished MCL activation in response to hostile jokes. These group differences may have important implications for our understanding of the neural correlates of social motivation and humor appreciation.

In recent years, increasing attention had been focused on gelotophobics (also called “gelatophobics”), that is, individuals who fear being the target of laughter. Gelotophobia, which was first defined as a pathological fear of laughter in 1995 based on Titze’s clinical observations^1^, is occasionally considered a type of social phobia, and gelotophobics show specific differences in social cognition. A salient behavioral feature of individual gelotophobics is a decreased subjective reward in response to social stimuli, which may present as a lack of interest in social interactions in general and less appreciation of humor in particular^2^. Gelotophobics suffer from a relatively high degree of social anxiety and have deficits in emotional regulation and an attention bias *away* from joyful laughter^3^. In addition, gelotophobics appear to be particularly sensitive towards “aggressive humor”^4^. It has been further argued that there may be a self-reinforcing effect at work because the tendency to appreciate humor less may lead gelotophobics to *appear* humorless, which may increase the likelihood of social isolation or, in the worst case, ridicule, leading to even more social anxiety and sensitivity towards aggressive humor^2^,^4^,^5^. Despite this growing understanding of gelotophobia, very little is currently known about the neural correlates underlying their responses to different types of jokes during humor appreciation. The present study seeks to contribute to our growing understanding of the neural correlates of personality, emotion, and social cognition^6^ by exploring the neural responses associated with hostile and non-hostile humor in gelotophobic individuals.

A broad understanding of the neural correlates of cognitive and affective processing of humor has emerged^7^,^8^,^9^,^10^,^11^, and distinct lines of research have begun to shed light on the particular neural patterns of behavior, such as violations of social norms^12^, processing differences between sexes/genders^13^ and responses to humor structures with different logical mechanisms^14^. However, the neural correlates of humor appreciation differ in not only the structure of humor but also the *content of humor*. A number of behavioral studies of humor appreciation have focused on humor with aggressive content^4^,^15^. However, surprisingly little is known about the neural reward mechanisms associated with the responses to aggressive humor or the underlying neural differences in the responses to aggressive humor among gelotophobics and non-gelotophobics.

Social motivation for goal-directed behavior depends on the expected value of the anticipated reward. The neural networks associated with social motivation consist of distinct ventral and dorsal systems. The *ventral* system is associated with the recognition of emotionally salient stimuli and the generation of emotional states (i.e., bottom-up emotional responses), whereas the dorsal system is involved in voluntary regulation of these states (i.e., voluntary top-down cognitive control of emotion)^16^,^17^,^18^. The ventral system comprises the amygdala, insula, ventral striatum (e.g., nucleus accumbens, NAcc), midbrain, ventral anterior cingulate cortex (vACC, BA 24/32/25), ventromedial prefrontal cortex (vmPFC, BA 10/11)/medial orbitofrontal cortex (OFC, BA 11/47), and ventrolateral prefrontal cortex (vlPFC, BA 45/47), whereas the *dorsal* system comprises the dorsal striatum (e.g., caudate), dorsal anterior cingulate gyrus (dACG), hippocampus and dorsal PFC (e.g., dlPFC and dmPFC, BA 9/44/46)^16^,^17^,^19^,^20^. A number of studies have demonstrated the self-regulation of emotion functions in specific brain areas, including the midbrain^21^, amygdala^22^,^23^, anterior insula^24^, anterior cingulate cortex (ACC)^25^, and inferior frontal gyrus (IFG)^26^.

It has been suggested that a lack of social motivation in major depressive disorder^20^ and autism^27^ might be attributed to dysfunction of the mesocorticolimbic (MCL) dopamine system. MCL dopamine circuits include a cognitive control circuit (PFC and ACC), a motivational and drive circuit (OFC/vmPFC), a memory and learning circuit (amygdala and hippocampus), and a reward circuit (ventral striatum and midbrain)^18^,^21^,^28^,^29^. The dopaminergic system is involved in reward encoding and reinforcement learning. Midbrain dopaminergic neurons fire in response to unexpected and highly motivating cues and are involved in reward expectancy and encoding and reinforcement learning^21^. The bilateral amygdalae have been shown to mediate the autonomic response to attachment insecurity in healthy humans^30^. Approach-avoidance dysfunction in anxiety disorders is focused in the amygdala, ventral striatum, insula, and PFC^31^. There is evidence for a general reward dysfunction in autism spectrum disorder (ASD). Specifically, the vACC and amygdala have been shown to display less activation within individuals with ASD in response to both social and monetary incentives^32^.

Humor is a social reinforcer and plays an important role in social perception and social incentives. Many fMRI studies of humor have associated humor appreciation and the experiencing of positive rewards with a central reward circuit in the ventral system^7^,^19^,^33^,^34^. Humor modulates the mesolimbic dopaminergic reward system, which includes the midbrain, NAcc, and amygdala^33^. Humor fMRI studies have shown that the midbrain contributes to ‘getting’ jokes and the subsequent experience of amusement^13^,^34^,^35^,^36^,^37^. Numerous behavioral studies have found that gelotophobics are more sensitive to social signals (e.g., aggressive humor, laughter, and facial expressions) than non-gelotophobics are^2^,^4^. Together, these findings suggest the possibility that dysfunctional reward processing in the ventral MCL dopaminergic reward system may be related to the social phobia or anxiety experienced by gelotophobics. At the same time, it is also known that the dlPFC plays a vital role in cognitive control processing, including decision-making and emotional regulation^18^.

The present study aimed to investigate the activation of the *ventral MCL dopamine reward system* in response to hostile and non-hostile jokes in gelotophobics compared to matched controls (non-gelotophobics). We predicted that gelotophobics would display greater activation of the *dorsal* system (e.g., dlPFC and dorsal stratum) for both hostile and non-hostile jokes, indicating higher levels of voluntary top-down cognitive control of emotion. Conversely, we expected that non-gelotophobics would show greater activation of the *ventral* system (MCL system, e.g., ventral striatum, midbrain, amygdala, vACC and ventral PFC) in response to both hostile and non-hostile jokes, indicating the generation and enjoyment of a positive emotional state. Additionally, we predicted that gelotophobics would exhibit dysfunctional activation of the ventral MCL system in response to both hostile and non-hostile jokes.

Unlike gelotophobics, who are less likely to enjoy humor, particularly aggressive humor, katagelasticists, individuals who enjoy laughing at others, enjoy aggressive humor^4^. Previous research has also shown that participants with high levels of hostility particularly enjoyed hostile humor^38^. In the present study, to focus on sensitivity or apprehension towards aggressive humor, we recruited gelotophobic and non-gelotophobic participants and excluded katagelasticists from both groups. We expected to observe dysfunction in the MCL system in both groups, particularly in gelotophobics, to *hostile jokes*. We further expected that non-gelotophobics would show stronger activation than gelotophobics would in the MCL system in response to *non-hostile jokes*. Finally, we employed psychophysiological interaction (PPI) analyses to confirm dysfunction in the MCL system in gelotophobics.

## Results

### Behavioral data

Participants rated the funniness of each condition on a 4-point scale (1 = not funny at all, 2 = not funny, 3 = funny, and 4 = very funny) during the scanning procedure. The mean funniness rating for the joke types was 3.01 ± 0.44 compared to the mean funniness rating of 1.80 ± 0.41 for the unfunny baseline stimuli. The interaction between the groups (gelotophobics and non-gelotophobics) and joke types (HJ, HS, NJ, and NS) on the funniness ratings was significant, *F*(3, 102) = 3.157, *p* = 0.028, *η*_p_^2^ = 0.085, and Bonferroni *post hoc* tests revealed that the two funny conditions were significantly funnier than the two unfunny conditions.

### fMRI results

#### Interactions between groups and joke types

**Figure 1:**
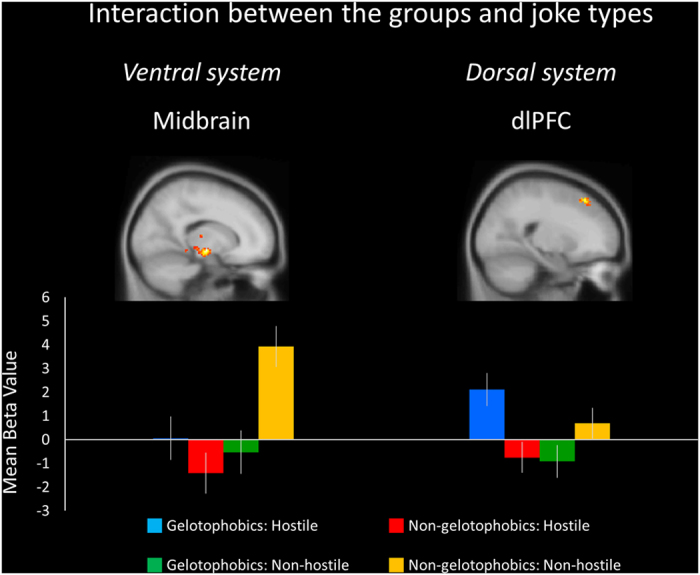
Interaction between the groups and joke types. The left midbrain shows significantly greater mean percent signal change values in the non-gelotophobics responding to non-hostile jokes. The right dorsolateral prefrontal cortex (dlPFC) displays greater mean percent signal change values in the gelotophobics for hostile jokes. Standard error of the mean (SEM) bars are shown.

There was an interaction between the groups and joke types in the ventral system (vlPFC, vmPFC, insula, and bilateral midbrain) and the dorsal system (bilateral dACC and bilateral dlPFC) (Table 1, Fig. 1). A *post hoc* test showed significant simple main effects for each of the different content types of the jokes between the gelotophobics and non-gelotophobics.

#### Atypical social brain responses in gelotophobics

##### Hostile joke types

**Figure 2:**
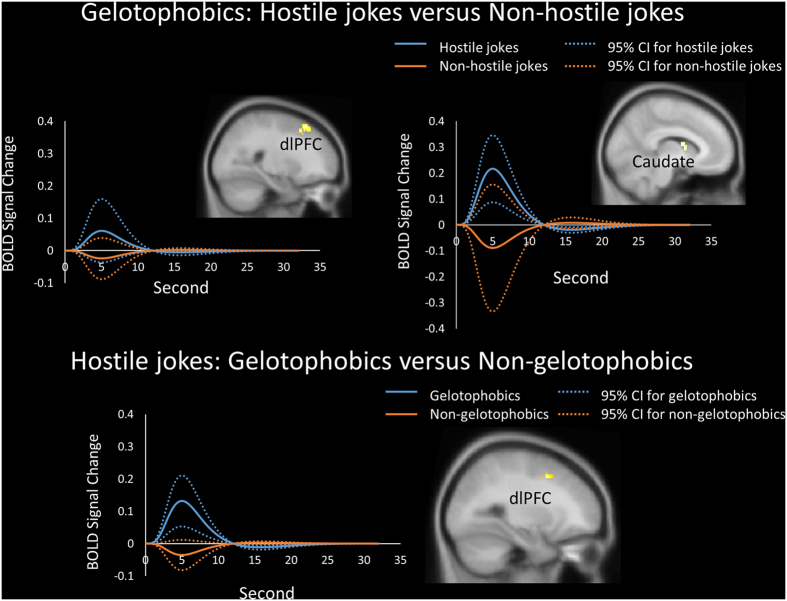
Gelotophobics responding to hostile jokes. Results showing the time course and average BOLD signals. (Top) Differences in the mean BOLD activation within the clusters across time in response to hostile jokes (blue) versus non-hostile jokes (orange) were found in gelotophobics in the right dorsolateral prefrontal cortex (dlPFC) and left caudate body (dorsal striatum). (Bottom) Gelotophobics (blue) showed greater activation in the left dlPFC than non-gelotophobics did (orange). The solid lines represent the evoked hemodynamic responses. The dotted lines represent the 95% confidence interval (95% CI) for the entire curve.

Joke type differences in the processing of hostile versus non-hostile jokes for gelotophobics were found in the right dlPFC, bilateral vlPFC, and left caudate body (dorsal striatum) (Table 2, Fig. 2). In terms of group differences for the hostile jokes (HJ-HS), gelotophobics showed greater activation in the left dlPFC than non-gelotophobics did (Table 3, Fig. 2).

##### Non-hostile joke types

Joke type differences in the processing of non-hostile jokes versus hostile jokes for gelotophobics showed greater activation in the bilateral caudate tail (Table 2). In terms of the group differences for the non-hostile jokes (NJ-NS), gelotophobics showed greater activation in the left caudate body (dorsal striatum) than non-gelotophobics did (Table 3).

#### Social brain activation and reward circuitry in non-gelotophobics

##### Hostile joke types

Joke type differences in the processing of hostile versus non-hostile jokes for non-gelotophobics were found in the right dlPFC, right vlPFC, and bilateral insula (Table 2). In terms of group differences for the hostile jokes (HJ-HS), non-gelotophobics showed greater activation in the left insula and right vlPFC than gelotophobics did (Table 3).

##### Non-hostile joke types

**Figure 3:**
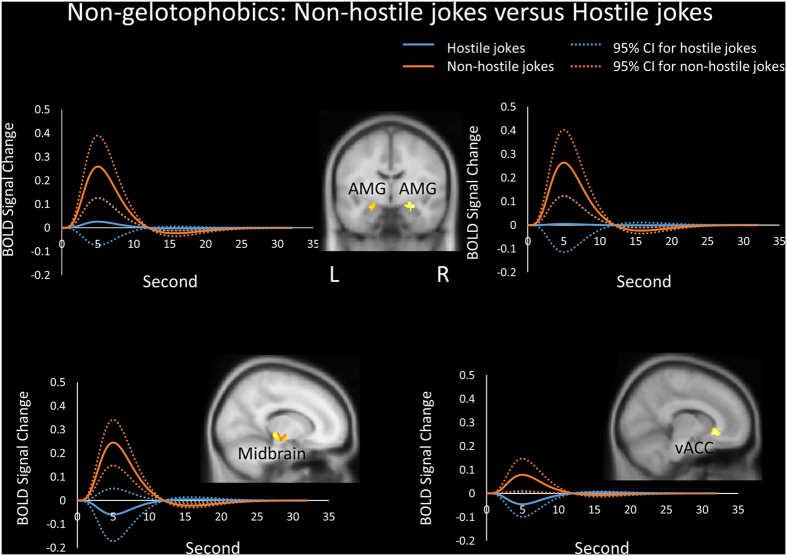
Non-gelotophobics responding to non-hostile jokes. Differences in the processing of non-hostile jokers versus hostile jokes in non-gelotophobics showed greater activation in the bilateral amygdalae, right midbrain (substantia nigra, SN), and right ventral anterior cingulate cortex (vACC). The solid lines represent the evoked hemodynamic responses. The dotted lines represent the 95% confidence interval (95% CI) for the entire curve. All figures are displayed in neurological convention. L = left; R = right.

Joke type differences in the processing of non-hostile jokers versus hostile jokes for non-gelotophobics were found in the form of greater activation in the left dACC, bilateral amygdalae, bilateral midbrain (substantia nigra, subthalamic nucleus, and medial geniculate body), right insula, right vACC, left vlPFC, left OFC and left NAcc (ventral striatum) (Table 2, Fig. 3).

**Figure 4:**
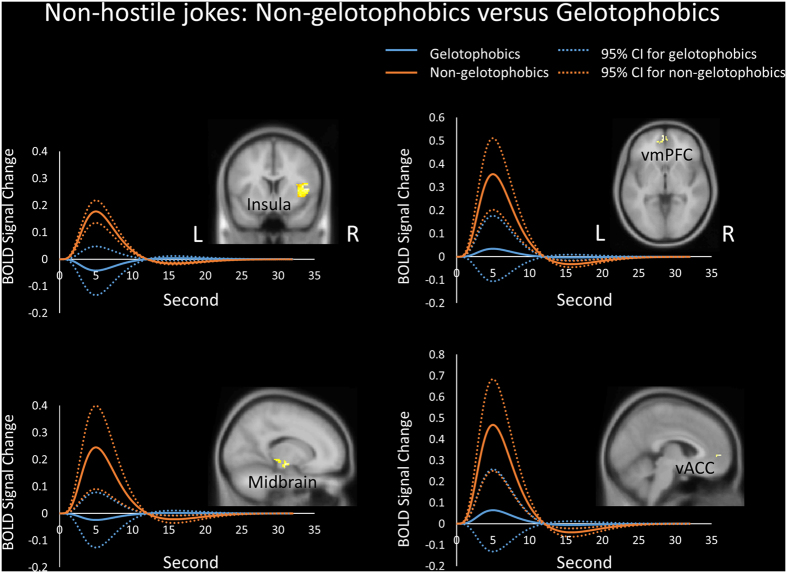
Group differences for the non-hostile jokes. Non-gelotophobics showed greater activation than gelotophobics in the bilateral insula, bilateral ventromedial prefrontal cortex (vmPFC), right midbrain (substantia nigra, SN), and left ventral anterior cingulate cortex (vACC). The solid lines represent the evoked hemodynamic responses. The dotted lines represent the 95% confidence interval (95% CI) for the entire curve. All figures are displayed in neurological convention. L = left; R = right.

In terms of group differences for the non-hostile jokes (NJ-NS), non-gelotophobics showed greater activation in the right insula, left vmPFC, right midbrain (substantia nigra, SN), and left vACC or pregenual ACC (pACC) than did the gelotophobics (Table 3, Fig. 4).

### Differences in evoked hemodynamic responses between the groups

To investigate differences between the groups in the time course of neural activation for the different joke types, we obtained time course data from the predefined ROIs in both the hostile and non-hostile conditions. The evoked hemodynamic responses, as measured by the average blood-oxygenation level dependent (BOLD) response, peaked at approximately 5 to 6 seconds after the stimulus onset and flattened at 12 seconds. Gelotophobics exhibited activation in the dlPFC and caudate in response to hostile stimuli (HJ-HS) versus non-hostile stimuli (NJ-NS). In terms of the hostile jokes (HJ versus HS), gelotophobics exhibited greater activation in the dlPFC than non-gelotophobics did (Fig. 2).

Furthermore, non-gelotophobics showed greater activation in the bilateral amygdalae, midbrain and vACC in response to non-hostile stimuli versus hostile stimuli. Non-gelotophobics also showed greater activation in these regions in response to non-hostile stimuli, whereas gelotophobics demonstrated little activation in the left amygdala and decreased activation in the midbrain and vACC (Fig. 3). Additionally, in terms of the non-hostile stimuli, non-gelotophobics showed greater activation in the insula, vmPFC, midbrain, and vACC than gelotophobics did (Fig. 4).

### Functional connectivity: Psychophysiological interaction analysis

**Figure 5:**
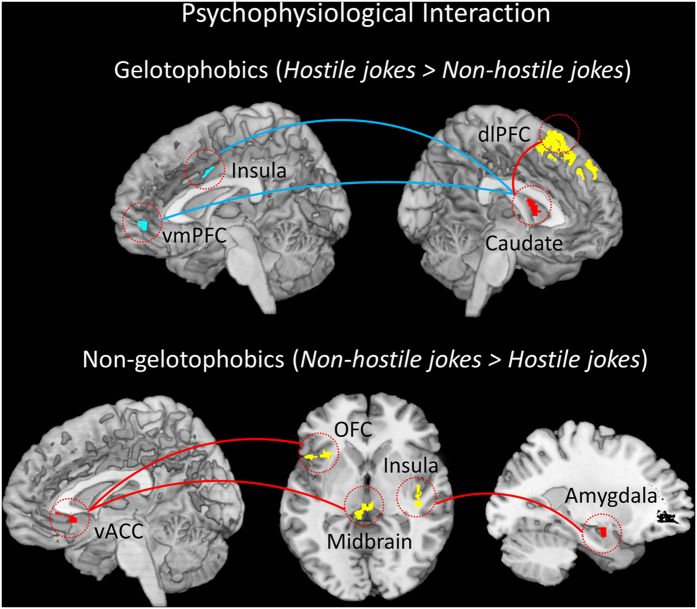
Results of the psychophysiological interaction analyses in gelotophobics and non-gelotophobics. (Top) Gelotophobics exhibited positive functional connectivity between the left caudate (seed) and the left dorsolateral prefrontal cortex (dlPFC), and negative connectivity between the right insula and right ventromedial PFC (vmPFC) for hostile jokes versus non-hostile jokes. (Bottom) Non-gelotophobics showed positive functional connectivity between the right amygdala (seed) and the right insula and positive functional connectivity between the right ventral anterior cingulate cortex (vACC) (seed) and the left midbrain and left orbitofrontal cortex (OFC). The red regions represent the seeds including the caudate, amygdala, and vACC. A red line represents a positive interaction, whereas a blue line represents a negative interaction.

The PPI analysis compared gelotophobics with non-gelotophobics in terms of the pattern of co-activation between different brain regions during humor appreciation. Gelotophobics exhibited BOLD responses in the left caudate (seed, region in red), *positive* increased functional coupling between the left caudate and the left dlPFC (red line), and a *negative* functional coupling between the right insula and the right vmPFC (blue line) (Table 4, Fig. 5, Top). Conversely, non-gelotophobics showed a positive functional connection between the right amygdala (seed) and the right insula, a positive functional connection between the left midbrain (seed) and the right caudate, a positive functional connection between the right vACC (seed) and the left midbrain and left OFC, and a positive functional connection between the left NAcc (seed) and the right caudate (Table 4, Fig. 5, Bottom). All red regions represent seeds.

**Figure 6:**
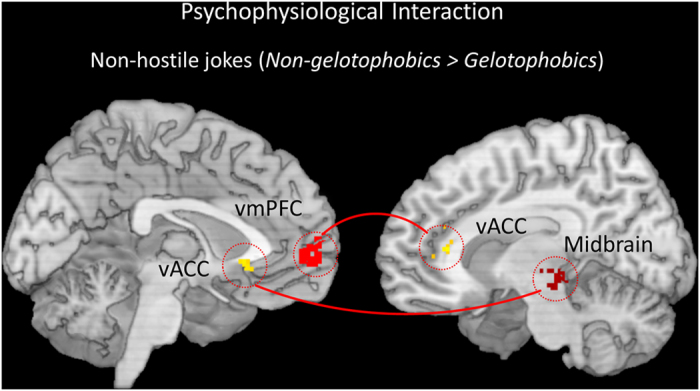
Results of the psychophysiological interaction analysis for non-hostile jokes. Compared with gelotophobics, non-gelotophobics showed increased coupling between the right midbrain and the left ventral anterior cingulate cortex (vACC) and increased coupling between the left ventromedial PFC (vmPFC) and the right vACC for non-hostile jokes. The red regions represent the seeds including the midbrain and vmPFC. A red line represents a positive interaction.

Group comparisons revealed no significant coupling between the left dlPFC (seed) and other brain regions (e.g., the MCL system) in gelotophobics compared with non-gelotophobics for the hostile jokes (HJ-HS). However, group comparisons did reveal significant positive coupling between the right midbrain (seed) and the left vACC and significant positive coupling between the left vmPFC (seed) and the right vACC in non-gelotophobics compared with gelotophobics for the non-hostile jokes (NJ-NS) (Table 4, Fig. 6).

## Discussion

The present study employed event-related fMRI to further advance our understanding of the neural correlates of responses to hostile and non-hostile jokes for gelotophobics and non-gelotophobics (with katagelasticists excluded from both groups). Gelotophobics showed greater activation than non-gelotophobics in the *dorsal corticostriatal system*, which comprises the dorsolateral prefrontal cortex and dorsal striatum (caudate). As expected, gelotophobics showed less activation in the ventral *mesocorticolimbic system* (MCL) in response to both joke types, particularly for hostile jokes. Conversely, non-gelotophobics displayed greater activation than gelotophobics did in the MCL system, particularly for non-hostile jokes.

The present study supports the hypothesis that gelotophobics involve greater activation in the dorsal corticostriatal system in response to both hostile and non-hostile jokes. The dorsal executive function neural circuit, which includes the dorsal regions of the dlPFC and dorsal striatum (caudate), is thought to modulate selective attention, planning, and effortful regulation of affective states^16^,^17^. The dorsal corticostriatal system, particularly the dorsal prefrontal feed-forward ‘action control’ and the dorsal striatal ‘habitual control’ system, appear to be involved in maintaining stable social attachments and preventing stress^39^. Activation of the dorsal executive and cognitive control circuitry (e.g., dlPFC and dorsal striatum) in gelotophobics may be related to enhancing cognitive control actions.

The dlPFC appears to play a vital role in the processing of decisions involving social preferences and possibly cognitive control^40^. Compared to healthy controls, anxious participants showed greater activity in the dlPFC, which suggested a control of emotion during down-regulation of negative emotions^41^. Gelotophobics showed greater engagement of the dlPFC than non-gelotophobics, perhaps suggesting a higher degree of cognitive control and emotional processing in response to hostile jokes.

PPI analyses demonstrated significantly greater functional connectivity in the dorsal corticostriatal system in gelotophobics in response to hostile jokes. Gelotophobics exhibited positive functional coupling between the left dorsal striatum (caudate) and the left dlPFC, suggesting the co-activation of the dorsal striatum and dlPFC changes significantly for hostile jokes versus non-hostile jokes. In addition, gelotophobics showed a significant negative connection between the dorsal striatum and the right insula and right vmPFC, suggesting a negative effect of coupling between the dorsal striatum and MCL as a function of the task.

The findings of the present study, however, which show greater activation in the dorsal corticostriatal system for gelotophobics, appear to be inconsistent with earlier research on schizophrenia. Dysfunction in reward anticipation and responses in schizophrenia have been linked to alterations in the corticostriatal system^42^. The role of corticostriatal interactions in mediating motivation and goal-directed behavior in gelotophobics and people with schizophrenia (with “negative symptoms”) should be investigated further.

The present study has taken an initial step to understand these interactions by investigating the dysfunction of the MCL reward system in gelotophobics for both hostile and non-hostile jokes. The present behavioral study showed that gelotophobics rated hostile jokes less funny than non-hostile jokes (see Supplementary information). These results appeared to be consistent with behavioral research on aggressive humor^4^. PPI analyses further confirmed dysfunction in the ventral MCL in gelotophobics compared to non-gelotophobics during humor appreciation of hostile jokes. Individuals in the gelotophobic group displayed no significant functional coupling between the left dlPFC and other brain regions of the MCL compared with the non-gelotophobic group. The findings of the present study suggest that the observed deficits in gelotophobics, in terms of humor appreciation, are associated with reduced function of the MCL reward system.

The MCL system is essential for cognitive and emotional brain functions and is involved in reward processing and in the mediation of amusement in response to affective stimuli^33^. Reward in humor appreciation is an important determinant of motivated behavior, and obtaining a reward is associated with feelings of amusement. The findings of the present study suggest that gelotophobics are more sensitive towards aggressive humor, which may lead gelotophobics to appreciate hostile humor less. Together, these results support the hypothesis that gelotophobics show significantly reduced coupling of the dorsal corticostriatal system with the mesolimbic dopaminergic reward system in response to both joke types during humor appreciation.

These findings may link the social withdrawal traits found in gelotophobics to earlier research on human anxiety disorders, including social phobia^43^,^44^, and mood disorders including depression^45^,^46^,^47^ and bipolar disorder (mania)^47^, which involve alterations in the dopaminergic system. Dysfunction of the mesolimbic dopamine system in the midbrain ventral tegmental area (VTA) and NAcc circuit are relevant to depression, which may include reduced motivation and decreased energy levels^46^. Additionally, impaired function in the subgenual ACC (sgACC) and other mesolimbic areas within this network could dysregulate emotion in individuals with depression or mania^47^. The mean gray matter volume of the sgACC cortex is abnormally reduced in mood disorders such as major depressive disorder (MDD) and bipolar disorder, which may explain their motivational and emotional manifestations^47^.

Conversely, we found markedly increased activation in the MCL reward system, including the midbrain, vmPFC, and vACC, in non-gelotophobics compared to gelotophobics, particularly for non-hostile jokes. As expected, we found greater activation in response to non-hostile jokes, which is consistent with the findings from previous studies showing activation of the MCL circuitry during anticipation of reward gain for non-hostile jokes^10^,^34^. PPI analyses further demonstrated significantly greater ventral MCL functional coupling between brain regions when watching non-hostile jokes versus hostile jokes in non-gelotophobics compared to gelotophobics. In particular, non-gelotophobics exhibited increased coupling between the right midbrain and the left vACC and increased coupling between the left vmPFC and the right vACC for non-hostile jokes, as evidenced by a significant group-by-type interaction in the MCL reward system.

The midbrain and ventral striatum appear to play a central role in the predictive neural coding of reward preference^48^. The dopamine midbrain system, which projects to the limbic system and PFC, plays a crucial part in many emotional and cognitive functions in the brain^49^. Humor fMRI studies have found that the midbrain contributes to ‘getting’ a joke and the subsequent positive experience of amusement^13^,^34^,^35^,^36^,^37^. Non-gelotophobics showed greater activation in the MCL reward circuitry, where dopamine in the midbrain serves as an important modulator by facilitating the consolidation of humor appreciation in ventral reactive reward system.

The findings of the present study contribute to a growing understanding of the neural correlates in response to both hostile and non-hostile jokes between gelotophobics and non-gelotophobics. However, some limitations of this study should be discussed. First, none of the participants were in the extreme gelotophobia category^2^, and most of the participants were in the slight gelotophobia category. Therefore, one limitation of this study was that few participants were recruited who had a “marked” fear of being laughed at. Second, most of the gelotophobics whom we recruited were college students. Therefore, external validation of the findings of this study to other groups such as workplace workers or clinical patients is another limitation of this study.

In conclusion, our results support the hypothesis that MCL dysfunction in gelotophobics occurs in response to both joke types, but particularly to hostile jokes. Gelotophobics showed greater activation than non-gelotophobics in the dorsal corticostriatal system, particularly the dlPFC, for hostile jokes. Non-gelotophobics showed greater activation than gelotophobics in the MCL system, including the midbrain, vmPFC, and vACC, particularly in response to non-hostile jokes. The MCL system may mediate the anticipation and experience of rewards^50^. Future studies should compare the neural correlates of gelotophobics with/without tendencies towards high hostility (e.g., gelotophobics with/without katagelasticists)^51^ in humor appreciation in response to hostile jokes that are based on psychoanalytic and superiority theories of humor. Additionally, future studies should further investigate the neural substrates of humor appreciation in gelotophobics and schizophrenics in the dorsal corticostriatal system.

## Methods

### Participants

Participants included 18 gelotophobics (11 men; mean age and SD = 23.72 ± 3.16; range, 20–30 years) and 18 matched non-gelotophobics (11 men; mean age and SD = 24.33 ± 3.31; range, 20–29 years), all of whom were right-handed, native Mandarin speakers with no history of neurological or psychiatric problems. All participants were evaluated with the traditional Chinese version of the PhoPhiKat-45, composed of three 15-item subscales measuring gelotophobia (Pho), gelotophilia (Phi), and katagelasticism (Kat) subscales^52^. Responses to the items were made on 4-point Likert-style scales. The empirically derived cut-off points were used to create the following categories: no (1.0–2.5), slight (2.5–3.0), marked (3.0–3.5) and extreme (3.5–4.0) fear of being laughed at^2^. A score of 2.5 was used as the cut-off point for identifying gelotophobic individuals, with participants scoring below 2.5 categorized as non-gelotophobics and participants scoring 2.5 or above categorized as gelotophobics. Most participants fell into the slight gelotophobia category, with almost none in the extreme gelotophobia category^53^. The exclusion criterion for katagelasticism was also set at 2.5, so that only candidates scoring less than 2.5 on the katagelasticism scale were included in the study. The mean gelotophobia rating was 2.89 ± 0.26 for gelotophobics and 2.03 ± 0.38 for non-gelotophobics. The mean katagelasticism rating was 2.03 ± 0.34 for gelotophobics and 1.86 ± 0.35 for non-gelotophobics (Table 5). All experiments of this study were approved by the Research Ethics Committee of National Tsing Hua University. I confirm that all of the participants gave their written informed consent and all experiments were performed in accordance with the relevant guidelines and regulations.

### Stimuli

Thirty-two hostile jokes (HJs) and 32 non-hostile jokes (NJs) were selected from an existing joke corpus and from previous studies^14^,^54^,^55^. Corresponding baseline stimuli were constructed by replacing the punch lines for all of these jokes with neutral stories of matching length and punctuation, resulting in 32 hostile sentences (HS) and 32 non-hostile sentences (NS). For the 64 jokes and 64 baseline stimuli, the setups were 75–89 characters in length (*M* = 78.59, *SD* = 4.14) and the punch lines were 15–20 characters in length (*M* = 18.00, *SD* = 1.91). Length and punctuation were matched across conditions for the setups and punch lines. The hostile jokes (HJ) and hostile baseline sentences (HS) were constructed using aggressive content. The non-hostile jokes (NJ) and non-hostile baseline sentences (NS) were constructed using non-aggressive content. The procedure for selecting the stimuli and the results are described in greater detail in supplementary data (see Supplementary Table S1).

### Experimental paradigm

**Figure 7:**
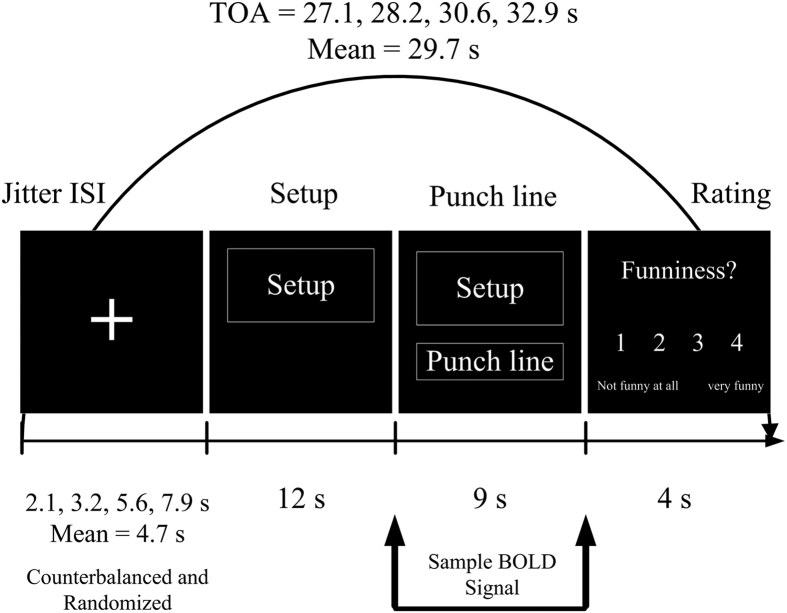
Timeline of the experimental trial within the scanner. Stimuli were presented in an event-related fMRI design with a trial-onset asynchrony (TOA).

The present study employed a 2 × 2 mixed factorial design, with group (gelotophobics versus non-gelotophobics) and joke type (hostile versus non-hostile) as factors, in an event-related fMRI paradigm using the E-Prime 2.0 software (Psychological Software Tools, Inc., Pittsburgh, PA, USA) to present the stimuli. The study examined the neural correlates in each group in response to two joke types and the corresponding baseline stimuli (HJ-HS and NJ-NS). During the scanning process, all stimuli were presented with white text on a black background. For each trial, a fixation cross appeared at a jittered inter-stimulus interval (ISI), which randomly varied between 2.1, 3.2, 5.6, and 7.9 s using a mathematical function and was counterbalanced across the stimulus types. The setup of the joke was shown for 12 s, after which the punch line was delivered and lasted for 9 s. The participants provided a subjective funniness judgment by pressing one of four buttons on a keypad positioned under their right hand to indicate how funny they thought the stimuli was (1 = ‘not funny at all’ to 4 = ‘very funny’), which lasted for 4 s (Fig. 7). The mean of the trial-onset asynchrony (TOA) was 29.7 s. There were four functional runs in total. Each functional run lasted 7 min and 55 s, with a 2-min break between runs. The total duration of the experiment was approximately 38 min and 5 s per participant.

### Image acquisition

T2*-weighted blood oxygenation level-dependent (BOLD) echo-planar images sequenced over the whole brain with 240 volumes were obtained using a Siemens Skyra 3T scanner (Erlangen, Germany) and a standard 32-channel head coil. Every volume contained 36 transverse slices (3.70-mm-thick, no gap) in an interleaved order that were obtained using the following acquisition parameters: TR = 2000 ms, TE = 30 ms, flip angle = 90°, 64 × 64 matrix, field of view (FOV) = 240 × 240 mm^2^, and voxel size = 3.75 × 3.75 × 3.70 mm^3^. High-resolution T1-weighted MPRAGE images of the entire brain were acquired using the pulse sequence: TR = 1900 ms, TE = 3.30 ms, flip angle = 9°, 256 × 256 matrix, FOV = 256 ×  256 mm^2^, voxel size = 1 × 1 × 1 mm^3^, and 192 1-mm thick contiguous axial images.

### Image analysis

Image processing and statistical analyses were carried out using Statistical Parametric Mapping software (SPM8; Wellcome Department of Cognitive Neurology, London, UK). For pre-processing, functional volumes for each participant were timing resliced, realigned, co-registered to the individual’s anatomical image, normalized to the standard Montreal Neurological Institute (MNI, McGill University, Montreal, Quebec, Canada) T1 template, and temporally high-pass filtered and spatially smoothed using a Gaussian kernel (FWHM = 8 mm).

After pre-processing, each participant’s BOLD signal was modeled with a fixed effects analysis that modeled the different event types (HJ, HS, NJ, and NS) for the punch line using a canonical hemodynamic response function (HRF) with a temporal derivative. Each participant was analyzed based on the a priori distinction between the jokes (funny) and the non-joke baseline stimuli (unfunny) using a general linear model (GLM) to perform within- and between-group comparisons. All six motion parameters were included in the GLM as nuisance regressors.

Each participant’s contrast volumes were fed into a random-effects analysis, which created group average maps for all contrasts across the entire brain using the flexible factorial design. Analysis of the parametric modulation were analyzed using an ANOVA, which allowed us to parse the main effect of joke type (HJ-HS vs NJ-NS), the main effect of group (gelotophobics vs non-gelotophobics), and the interactions between the group and joke type.

A region of interest (ROI) statistical analysis was performed for a specific a priori hypothesis. Anatomical ROI maps were generated using WFU PickAtlas Tool software (www.fmri.wfubmc.edu) that generates ROI masks. Based on previous studies^20^,^27^,^32^, the resulting mask of humor appreciation was associated with brain regions in the predefined ROI. Specifically, the analyses of the ventral and dorsal systems focused on seven masks in the midbrain, ventral striatum (NAcc), amygdala, insula, anterior cingulate cortex (ACC), prefrontal cortex (PFC), and dorsal striatum (e.g., caudate nucleus).

Our main goal was to investigate the time course of neural activation in the dorsal corticostriatal system and MCL system in response to humor appreciation for different joke types between the groups. We further extracted the average time course for the different types of jokes between the groups. We used default canonical HRF in SPM to estimate the evoked BOLD responses. The default canonical HRF used 2 gamma functions to simulate the evoked BOLD responses, which peaked approximately 6 s after the event and was followed by an undershoot. However, this function did not simulate the initial dips of 1 to 2 s after the stimulation. The plot, in terms of the fitted response and peri-stimulus time histograms (PSTH), was the average response to an event with a mean signal ± SE for each peri-stimulus time bin. Error bars indicate the 95% confidence interval.

Finally, the present study also conducted psychophysiological interaction (PPI) analyses to investigate functional connectivity within the dorsal corticostriatal system (dlPFC and caudate), which mediates the top-down social cognitive control of emotion, and the MCL reward system (midbrain, NAcc, amygdala, vACC, vmPFC), which mediates bottom-up emotional experiences of affective amusement.

The present study performed two PPI analyses to focus on within- and between-group differences in functional connectivity between brain regions related to changes in the psychological variables (e.g., hostile jokes and non-hostile jokes). PPI are regression-based connectivity analyses that examine changes in the contribution of activation in one brain region (i.e., seed region) to another region based on changes in the psychological context^56^. For each participant and seed region, the first eigenvariate time course of the seed volume of interest was extracted from a 10-mm radius sphere around the center of the predetermined coordinates as implemented in SPM8 in the first level. The time course of the BOLD signal corresponding to each of the eight regions of interest (seeds) were deconvolved to generate the time course of the neuronal signal. The time course of the neuronal signal for the responses to the two joke types (funny and unfunny) was created, which resulted in a psychological vector (Y regressor) representing the time course of the eight seed regions, a psychological vector (P regressor) representing the contrast of the two joke types (e.g., hostile versus non-hostile jokes), and their interaction (PPI regressor) representing the interaction between the physiological and psychological factors. After convolution with the canonical HRF, the three regressors (i.e., PPI, P, and Y) for each functional run (run-by-run) and the effects of no interest (i.e., six motion correction parameters) were entered into a signal first-level GLM. The PPI analyses were then entered into a second-level random effects group analysis using a one-sample *t*-test for each group separately and a two-sample *t*-test for group comparisons.

Both subtraction and functional connectivity analyses were undertaken using a ROI approach. The threshold of activation of the predefined ROIs were set at a voxel-wise *p* < 0.05 FWE (family-wise error rate) for multiple comparisons with eight contiguous voxels using a small volume correction (SVC) and a 10-mm sphere on the coordinates of interest. All figures are shown in neurological convention (participants’ left is displayed on the left).

## Additional Information

**How to cite this article**: Chan, Y.-C. Neural Correlates of Deficits in Humor Appreciation in Gelotophobics. *Sci. Rep.* **6**, 34580; doi: 10.1038/srep34580 (2016).

## Supplementary Material

Supplementary Information

## Figures and Tables

**Table 1:** Brain regions associated with the interactions between group and joke type.

Anatomical region	BA	Voxels	Side	MNI coordinates			Z score
				*x*	*y*	*z*	
***Ventral system***							
Inferior frontal gyrus (vlPFC)	46	59	L	−42	42	12	4.61
Medial frontal gyrus (vmPFC)	10	46	L	−2	60	−2	4.41
Insula	13	110	R	40	−6	−2	4.30
Midbrain (subthalamic nucleus)	—	11	L	−8	−10	−8	3.99
Midbrain (substantia nigra)	—	37	R	16	−22	−8	3.80
***Dorsal system***							
Anterior cingulate cortex (dACC)	32	28	L	−18	34	18	4.92
Anterior cingulate cortex (dACC)	32	131	R	12	38	14	4.24
Superior frontal gyrus (dlPFC)	8	13	R	22	26	52	3.81
Superior frontal gyrus (dlPFC)	8	13	L	−22	28	50	3.67

The activation threshold for the simple main effects was set at *p* < 0.05 FWE (family-wise error rate) for multiple comparisons using predefined ROIs for each type of joke. vlPFC = ventrolateral prefrontal cortex; vmPFC = ventromedial PFC; dACC = dorsal anterior cingulate cortex.

**Table 2:** Within-group comparisons in brain regions differentially activated for the simple main effects.

Anatomical region	BA	Voxels	Side	MNI coordinates			Z score
				*x*	*y*	*z*	
**Gelotophobics** (**hostile type** > **non-hostile type**)							
Superior frontal gyrus (dlPFC)	8	128	R	30	20	52	4.44
Inferior frontal gyrus (vlPFC)	46	36	L	−48	42	12	4.14
Inferior frontal gyrus (vlPFC)	45	24	R	50	16	16	4.08
Caudate body (dorsal striatum)	—	38	L	−8	0	12	3.63
**Gelotophobics** (**non-hostile type** > **hostile type**)							
Caudate tail	—	74	R	36	−46	8	5.20
Caudate tail	—	43	L	−38	−46	8	4.29
**Non-gelotophobics** (**hostile type** > **non-hostile type**)							
Middle frontal gyrus (dlPFC)	9	113	R	40	16	36	5.92
Middle frontal gyrus (vlPFC)	10	34	R	32	60	6	4.74
Insula	47/13	21	R	30	20	−4	4.94
Insula	13	21	L	−32	22	6	3.88
**Non-gelotophobics** (**non-hostile type** > **hostile type**)							
Anterior cingulate cortex (dACC)	32	106	L	−18	36	20	6.17
Amygdala	—	56	R	22	−10	−12	5.72
Midbrain (substantia nigra)	—	181	R	14	−24	−8	5.58
Insula	13	28	R	30	−32	20	5.29
Anterior cingulate cortex (vACC)	32	87	R	10	26	−6	5.00
Midbrain (subthalamic nucleus)	—	63	L	−12	−12	−8	4.85
Amygdala	—	40	L	−22	−10	−12	4.52
Midbrain (medial geniculate body)		46	L	−20	−24	−6	4.43
Inferior frontal gyrus (vlPFC)	46	12	L	−36	36	14	4.31
Orbitofrontal cortex (OFC)	47	13	L	−32	32	−16	4.13
Nucleus accumbens (NAcc)	—	39	L	−4	0	−4	3.55

The activation threshold for the simple main effects was set at *p* < 0.05 FWE (family-wise error rate) for multiple comparisons using predefined ROIs for each type of joke. dlPFC = dorsolateral prefrontal cortex; vlPFC = ventrolateral PFC; vmPFC = ventromedial PFC; vACC = ventral anterior cingulate cortex; dACC = dorsal ACC.

**Table 3:** Between-group comparisons in brain regions differentially activated for the simple main effects.

Anatomical region	BA	Voxels	Side	MNI coordinates			Z score
				*x*	*y*	*z*	
**Hostile type** (**gelotophobics** > **non-gelotophobics**)							
Middle frontal gyrus (dlPFC)	8	16	L	−26	10	46	4.38
**Non-hostile type** (**gelotophobics** > **non-gelotophobics**)							
Caudate body (dorsal striatum)	−	8	L	−12	2	26	3.54
**Hostile type** (**non-gelotophobics** > **gelotophobics**)							
Insula	13	14	L	−36	18	10	3.42
Inferior frontal gyrus (vlPFC)	45/47	13	R	34	26	10	3.32
**Non-hostile type** (**non-gelotophobics** > **gelotophobics**)							
Insula	13	183	R	46	10	6	5.16
Medial frontal gyrus (vmPFC)	10	59	L	−6	60	−2	4.10
Midbrain (substantia nigra)	—	60	R	12	−26	−6	3.93
Anterior cingulate cortex (vACC)	32/10	21	L	−6	50	0	3.75

The activation threshold for the simple main effects was set at *p* < 0.05 FWE (family-wise error rate) for multiple comparisons using predefined ROIs for each type of joke. dlPFC = dorsolateral prefrontal cortex; vlPFC = ventrolateral PFC; vmPFC = ventromedial PFC; vACC = ventral anterior cingulate cortex.

**Table 4:** Functional connectivity of the psychophysiological interaction analyses.

Anatomical region	BA	Voxels	Side	MNI coordinates			Z score
				*x*	*y*	*z*	
**Gelotophobics** (**hostile type** > **non-hostile type**)							
*Seed: Caudate body (−8*, *0*, *12*) *Positive connection*							
dlPFC	8	78	L	−16	22	52	3.48
*Seed: Caudate body (−8*, *0*, *12*) *Negative connection*							
Insula	13	45	R	30	−6	28	3.84
vmPFC	10	19	R	4	54	−4	2.93
**Non-gelotophobics** (**non-hostile type** > **hostile type**)							
*Seed: Amygdala (−22*, *−10*, *−12*) *Positive connection*							
Insula	13	17	R	42	−26	−4	3.44
*Seed: Midbrain (−12*, *−12*, *−8*) *Positive connection*							
Caudate body	—	32	R	18	2	22	3.06
*Seed: vACC (10*, *26*, *−6*) *Positive connection*							
Midbrain	—	80	L	−8	−28	−8	3.70
OFC	47	38	L	−32	20	−2	2.97
*Seed: NAcc (−4*, *0*, *−4*) *Positive connection*							
Caudate	—	10	R	10	10	8	2.96
**Hostile type** (**gelotophobics** > **non-gelotophobics**)							
*Seed: dlPFC (−26*, *10*, *46*) *Positive connection*							
*n*.*s*.							
**Non-hostile type** (**non-gelotophobics** > **gelotophobics**)							
*Seed: Midbrain (12*, *−26*, *−6*) *Positive connection*							
vACC	23	24	L	−6	22	−4	3.94
*Seed: vmPFC (−6*, *60*, *−2*) *Positive connection*							
vACC	45	24	R	12	32	10	3.53

The activation threshold for the PPI analysis was set at *p* < 0.05 FWE (family-wise error rate) using predefined ROIs. dlPFC = dorsolateral prefrontal cortex; vmPFC = ventromedial PFC; OFC = orbitofrontal cortex; NAcc = Nucleus accumbens; *n*.*s*. = not significant; vACC = ventral anterior cingulate cortex.

**Table 5:** Range, mean, and standard deviations for gelotophobics and non-gelotophobics in the gelotophobia and katagelasticism subscales.

	gelotophobia subscale			katagelasticism subscale		
	range	*M*	*SD*	range	*M*	*SD*
Gelotophobics	2.60 ~ 3.47	2.89	0.26	1.40 ~ 2.47	2.03	0.34
Non-gelotophobics	1.13 ~ 2.47	2.03	0.38	1.07 ~ 2.47	1.86	0.35
