# Supplementary material for: Neural Correlates of Deficits in Humor Appreciation in Gelotophobics
Source: Sci Rep. 2016 Oct 3;6:34580. doi: 10.1038/srep34580 (PMC5046107; doi:10.1038/srep34580)
Supplement: Supplementary Information [file srep34580-s1.pdf]

# Neural Correlates of Deficits in Humor Appreciation in Gelotophobics

Yu-Chen Chan*

Institute of Learning Sciences, National Tsing Hua University, Hsinchu, Taiwan

## Supplementary Information

To ensure that the jokes were valid as stimuli, two behavioral studies were conducted prior to the fMRI experiment. In the first preliminary study, 44 hostile jokes (HJs) and 32 non-hostile jokes (NJs) were selected from an existing joke corpus and from previous studies.[14,58,59] Fifty-four participants (36 men, ranging from 18 to 24 years-old, aged 20.41 ± 1.46 years) rated each joke on its degree of comprehensibility, funniness, and hostility, using a 7-point scale. Thirty-six HJs with ratings above 4 for both funniness and hostility were selected. For the 68 jokes of both types remaining, the mean comprehensibility rating was 6.88 ± 0.18, and the mean funniness rating was 4.61 ± 0.40. A one-way repeated-measures ANOVA revealed that the two types of jokes did not differ significantly in terms of funniness ($F(1, 53) = 2.038$, $p = 0.159$, $\eta_p^2 = 0.04$). The mean hostility rating was 5.07 ± 0.49 for the hostile jokes and 1.36 ± 0.31 for the non-hostile jokes, a difference found to be significant using a one-way repeated-measures ANOVA ($F(1, 53) = 603.407$, $p < 0.001$, $\eta_p^2 =$

0.92).

A second study was conducted with a separate group of 20 gelotophobics (mean gelotophobia score = 2.75 ± 0.21, ages from 19 to 23 years-old, mean = 20.75 ± 1.59 years) and 20 non-gelotophobics (mean gelotophobia score = 2.00 ± 0.31; ages from 18 to 30 years-old, mean = 20.70 ± 2.66 years). The 36 HJs and 32 NJs were used. Corresponding base-line trials were constructed by replacing the punch lines for all of these jokes with neutral stories of matching length and punctuation, resulting in 36 hostile sentences (HS) and 32 non-hostile sentences (NS). All participants viewed and rated each trial using E-Prime software on its degree of comprehensibility, funniness and hostile on a 7-point scale. Based on the results of this behavioral study, the present study selected the 32 most salient HJs. The mean and standard deviation for comprehensibility was 6.30 ± 0.82, indicating that all stimuli were comprehensible to participants. The means and standard deviations for the rated levels of funniness and hostility for all four conditions of 32 stimuli each are shown in Table S1.

A two-way mixed ANOVA was conducted to investigate the influence of group (gelotophobics and non-gelotophobics) and joke type (HJ, HS, NJ, and NS) on funniness and hostility ratings. There was a main effect of type on funniness rating, $F(3, 114) = 173.289$, $p < 0.001$, $\eta_p^2 = 0.820$, indicating that jokes in the funny condition (HJ and NJ) were significantly funnier than those in the unfunny conditions

(HS and NS). There was no significant group and type interaction, $F(3, 114) = 1.736$, $p = 0.164$, $\eta_p^2 = 0.044$. Likewise, There was a main effect of joke type on hostility rating, $F(3, 114) = 242.792$, $p < 0.001$, $\eta_p^2 = 0.865$, indicating that the two hostile conditions (HJ and HS) were rated as being significantly more hostile than the two non-hostile conditions (NJ and NS). There was no significant group and type interaction, $F(3, 114) = 0.329$, $p = 0.805$, $\eta_p^2 = 0.009$. Additionally, in order to compare the two joke types between groups, a two-way mixed ANOVA with group (gelotophobics and non-gelotophobics) and joke types (HJ and NJ) was also performed on funniness ratings. There was no main effect of group, $F(1, 38) = 1.190$, $p = 0.282$, $\eta_p^2 = 0.030$. There was a significant group and type interaction, $F(1, 38) = 5.669$, $p = 0.022$, $\eta_p^2 = .130$. In gelotophobics, non-hostile jokes were rated as funnier than hostile jokes, $F(1, 38) = 16.589$, $p < 0.001$, $\eta_p^2 = 0.304$.

For the 64 jokes and 64 baseline stimuli, the setups were 75-89 characters in length ($M = 78.59$, $SD = 4.14$) and the punch lines were 15-20 characters in length ($M = 18.00$, $SD = 1.91$). Length and punctuation were matched across conditions for the setups and punch lines. The hostile jokes (HJ) and baseline sentences (HS) were constructed using aggressive content. For example, one hostile stimulus pair read:

At one time, there were street gangs in Taipei whose members would

randomly toss acid into the faces of young ladies walking in the street. One day, a young gangster was sneaking up from behind a woman when she suddenly turned around. He saw her face and said…

Punchline in funny condition (HJ): “Nevermind, we already got her!”

Punchline in unfunny condition (HS): “I’ve never seen such an ugly lady.”

The non-hostile jokes (NJ) and baseline sentences (NS) were constructed using non-aggressive content. For example:

Shawn and Jane were a hardworking couple, trying to build a fortune together from scratch. One day, Jane asked, “Why can’t we live in a more expensive apartment?” Shawn replied, “Don’t worry! Your wish is going to come true.” Jane exclaimed, “Really? That’s fantastic!”

Punchline in the funny condition (NJ): Shawn said, “Yes, our landlord is raising the rent.”

Punchline in the unfunny condition (NS): Shawn said, “Yes, I’m preparing a new housing budget.”

Table S1 Mean and standard deviation for two groups in four conditions for funniness and hostility ratings

| | Gelotophobics | | | | Non-gelotophobics | | | |
|---|---|---|---|---|---|---|---|---|
| | Funniness | | Hostility | | Funniness | | Hostility | |
| | *M* | *SD* | *M* | *SD* | *M* | *SD* | *M* | *SD* |
| HJ | 4.23 | 1.06 | 4.73 | 1.14 | 4.88 | 1.28 | 4.45 | 1.30 |
| HS | 2.50 | 0.75 | 4.78 | 1.20 | 2.77 | 0.91 | 4.68 | 1.24 |
| NJ | 4.86 | 1.08 | 1.87 | 0.70 | 4.98 | 1.29 | 1.49 | 0.50 |
| NS | 2.40 | 0.77 | 1.88 | 0.68 | 2.48 | 0.93 | 1.56 | 0.47 |

HJ = hostile jokes; HS = hostile sentences; NJ = non-hostile jokes; NS = non-hostile sentences
